# P-1863. Maternal Mycobacterial-Specific T-cell Signatures and Infant Risk of *Mycobacterium tuberculosis* Infection

**DOI:** 10.1093/ofid/ofae631.2024

**Published:** 2025-01-29

**Authors:** Talia Himmelfarb, Alex Warr, John Kinuthia, Daniel Matemo, Jerphason O Mecha, Sylvia M LaCourse, Grace John-Stewart, Whitney Harrington, Thomas Hawn

**Affiliations:** University of Washington, Seattle, Washington; Emory University, Atlanta, Georgia; Kenyatta National Hospital, Nairobi, Nairobi Area, Kenya; Kenyatta National Hospital, Nairobi, Nairobi Area, Kenya; Kenyatta National Hospital, Department of Obstetrics and Gynaecology, Nairobi, Kenya, sotik, Nyanza, Kenya; University of Washington, Seattle, Washington; University of Washington, Seattle, Washington; University of Washington / Seattle Children's Research Institute, Seattle, Washington; University of Washington, Seattle, Washington

## Abstract

**Background:**

Tuberculosis disease (TB) caused 214,000 pediatric deaths in 2022. A growing body of evidence suggests that HIV exposed uninfected infants (iHEU) are at increased risk for *Mycobacterium tuberculosis* (Mtb) infection. Harnessing the power of the maternal immune system to protect infants has shown promise in other infections. Yet no well powered study has evaluated the association between maternal mycobacterial-specific T cell memory and infant protection from Mtb infection. To address this knowledge gap, we examined the hypothesis that previously undescribed maternal factors modulate infant immunity to bacillus Calmette-Guèrin (BCG) and Mtb.

**Fig 1.**
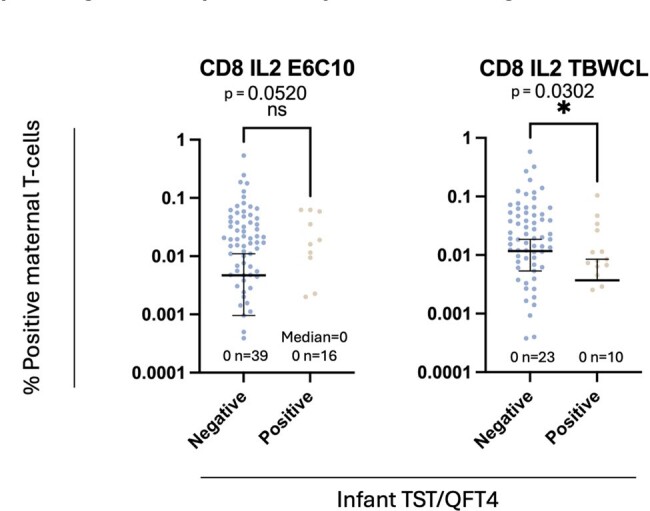
Mothers of infants negative for Mtb infection had higher proportions of CD8 T cells expressing IL2 in response to mycobacterial antigens. ESAT-6/CFP-10 negative median 0.004710 (95% CI 0.00096000-0.01103), positive median 0.0 (95% CI 0-0.009410). TBWCL negative median 0.01172 (95% CI 0.005340-0.01860), positive median 0.003710 (95% CI 0-0.008467).

**Methods:**

This study was nested within a randomized controlled trial of isoniazid to prevent Mtb infection in 300 iHEU (iTIPS) who were vaccinated with BCG at birth, randomized to isoniazid or placebo at 6-10 weeks, and had Quantiferon-Plus (with IFNγ, IL2, IP10, TNF measured in supernatant) (QFT-4) at 14 months, and/or tuberculin skin testing (TST) at 14 and 24 months. Paired maternal peripheral blood mononuclear cells were collected at the 6-10 week visit. The majority (73%) of mothers started antiretroviral therapy before pregnancy, 26.3% during pregnancy, and 0.7% after pregnancy. Frequency of self-reported history of TB disease was 10.7%. We assessed the response of maternal T cells to Mtb whole cell lysate (TBWCL), a proxy for BCG, and ESAT-6/CFP-10 (E6C10), an Mtb specific peptide pool, by flow cytometry with a T cell panel including surface, memory, and activation markers and IL2, IL17A, IFNγ, and TNF.

**Results:**

Our preliminary analysis of 166 of 235 maternal samples with flow cytometry revealed that mothers of infants with positive TST or QFT-4 had significantly fewer CD8^+^IL2^+^ cells in response to TBWCL (median 0.0037% vs 0.0117%, p=0.032) (Fig 1). Conversely mothers of these infants had significantly higher CD4^+^TNFa^+^ responses to E6C10 (median 0.0458% vs 0%, p=0.0014) and CD4^+^IFNγ^+^ responses to TBWCL (median 0.0533% vs 0.0302%, p=0.0395).

**Conclusion:**

These results establish that maternal mycobacterial-specific immune signatures are associated with infant outcomes, which we plan to further investigate by comparing these maternal signatures with infant T cell response to TBWCL and determining the role of maternal microchimeric cells.

**Disclosures:**

Sylvia M. LaCourse, MD, MPH, Merck: Grant/Research Support|UpToDate: Royalties

